# Prevalence of HIV Infection and Associated Risk Factors among Men Who Have Sex with Men (MSM) in Harbin, P. R. China

**DOI:** 10.1371/journal.pone.0058440

**Published:** 2013-03-13

**Authors:** Ling Zhang, Dandan Zhang, Baowen Yu, Shangbo Wang, Yanlin Liu, Jian Wang, Xin Li, Xiaoyun Shang, Hongyuan Li

**Affiliations:** 1 Department of Epidemiology, Public Health College, Harbin Medical University, China; 2 Antituberculosis Station, Harbin, China; 3 Sanitation Supervision Stations, Harbin, China; 4 Centers for Disease Control and Prevention, Harbin, China; Fundacion Huesped, Argentina

## Abstract

**Objective:**

To assess the prevalence of HIV infection and characteristically risk of factors which associated with HIV infection among MSM in Harbin, China.

**Methods:**

A face-to-face questionnaire interview was conducted among 463 Men Who Have Sex with Men (MSM) who were recruited by the snowball sampling in Harbin from April, 2011 to July, 2011. The questionnaire mainly included demographics, AIDS knowledge, homosexual behavior and the status of intervention in MSM. Blood specimens were obtained and tested for the diagnoses of HIV, syphilis and hepatitis C virus (HCV). Associations between above exposed factors and HIV infection were analyzed using a univariate analysis and forward stepwise logistic regression.

**Results:**

The prevalence of HIV and syphilis was 9.5 and 14.3%. The awareness rate of AIDS was 86.8%. The rate of unprotected sexual behavior was 57.6% of MSM during the past 6 months. The univariate analysis identified that the age (age≥35 years old), cohabitation, more than 10 years of homosexual behavior and more than 10 homosexual partners were risk factors which associated with the HIV infection, and that protected sex during the past 6 months was a protective factor for the HIV infection. The multivariate analysis identified that the duration of homosexual behavior and commercial sexual behavior were independent risk factors which associated with the HIV infection, and the protected sex during the past 6 months was a protective factor for the HIV infection.

**Conclusion:**

The prevalence of HIV among MSM in Harbin has been rapidly increasing in the past few years. Targeted, tailored, and comprehensive interventions are urgently needed to prevent the HIV infection from MSM.

## Introduction

The HIV/AIDS (Acquired Immune Deficiency Syndrome, AIDS) epidemic has evolved to become the greatest challenge in global health since first recognition in 1981[1]. The World Health Organization (WHO) estimated that there would be 34.2 million adults and children worldwide living with HIV/AIDS, which resulted in about 1.7 million deaths in 2011 (UNAIDS 2012). AIDS has claimed more than 25 million lives and more than 60 million people have become infected with HIV. By now, more than 7,000 people per day including 1,000 children are newly infected with HIV (UNAIDS 2012).

The first AIDS patient in the world was found in male homosexuality, and firstly widespread in the crowd. Men who have sex with men (MSM) contain heterosexuals and bisexuals who have sexual contacted with male. MSM commonly have multiple sex partners and higher proportion of unprotected sex behavior compared with non-MSM male, suggesting that they are a high risk population for HIV transmission [2]–[3]. In 2009, MSM accounted for 61% of new infection in the United States [4]. HIV prevalence among MSM was 38 times higher than that of the general population in Nicaragua in 2011 [5].

In China, urbanization accelerated by economic growth since the 1980s, there has a complicated task for the prevention of HIV/AIDS. In 2006, the UNAIDS (The Joint United Nations Program on HIV and AIDS) indicated a growing evidence of HIV outbreaks among Chinese MSM (UNAIDS 2006). Up to now, MSM have become one of the priority populations for prevention and control of HIV pandemic in China. Although MSM was accounted for only 2–4% of the Chinese adult male population [6], it was estimated that 77,000 MSM were living with HIV/AIDS and accounted for 11% of the total number of estimated HIV cases in China by the end of 2007, and only 0.4% in 2005 [6]–[7]. In addition, MSM represented an increasing proportion of new HIV infections in China: 0.2% in 2001, 7.3% in 2005, 12.2% in 2007, and 32.5% in 2009 [8]–[11]. Based on these data, we will know that estimated cases or new cases were increasing the proportion of MSM population yearly.

The data from a large-scale national survey from 18,000 MSM conducted across 61 cities of China indicated an overall prevalence of 4.9% [12], with four cities reporting an HIV prevalence exceeding 10% in 2008–2009 [13]. Several previous studies have been shown that the prevalence of HIV is one of the highest in MSM and related populations in metropolitan areas such as Guangzhou, Shanghai and Beijing [14]–[17] [18]. It indicated a steady increase in HIV prevalence among MSM [19]–[22]. It is important to curb the spread of HIV epidemic in this kind of vulnerable population in China.

Harbin is the economic and commercial centers of Heilongjiang province in northeast of China. In a previous study, the rate of HIV infections in Harbin MSM showed an increasing trend from 1% in 2006 [23] to 4.4% in 2008 [24]. However, the relevant status of HIV infections in MSM is still unclear in Harbin. In order to prevent the epidemic of HIV infection, it is very important to understand the prevalence of HIV and risk factors of HIV infection in MSM in Harbin, P. R. China. Thus, the objective of this study was to monitor the current rate of HIV epidemic, to interfere the population at the risk of HIV infections, and to evaluate the effectiveness of prevention programs among MSM in Harbin, P. R. China. It will be essential to obtain current data about the HIV prevalence and the risk factors which associated with recent HIV infection amongst MSM in Harbin, P. R. China.

## Materials and Methods

### Participant's enrolment

Snowball sampling was used in this study. The participant's recruitment in details was as follows: through the recommendation of NGO, some male volunteers should meet our requirements: i.e. over 18 years old, and had sexual behavior with men, including oral and anal sex in the last 12 months. We selected the seeds according to the type of activity places, and encouraged the seeds to drive the same type companions to take part in our investigation. The companions also can drive his companions after his investigation. Thus, 463 samples were collected during April, 2011 and July, 2011.

The review board of Harbin Public Health Bureau issued a formal written waiver for the need of ethics approval. Written informed consent was obtained from all participants.

### Demographics, homosexual behavior and the status of intervention

Data were collected on demographics, AIDS awareness, sexual behavior, status of intervention and venereal disease in the standardized questionnaires that were administrated through face-to-face interviews by the trained physicians. Blood specimens were collected from participants and tested for diagnoses of HIV, syphilis and hepatitis C virus (HCV). The contact information including the mobile phone number was obtained from each participant. All participants were trained the general information about HIV and syphilis and informed how to practice safe sex through a pre-test and post-test counseling provided by this study. Each participant was given bus transportation allowance. The test results of each participant were to be sent in a short message to their mobile phone, and the content of message should include the advice how to be far away from high-risk sex behavior.

### Laboratory testing

Blood samples were tested for antibodies of HIV, syphilis and HCV. The screening tests for antibodies of HIV, syphilis and HCV, and WB (Western blot assay, WB) were conducted at Harbin CDC, P. R. China. The presence of HIV-1 antibody was tested by ELISA (enzyme-linked immunosorbent assay, ELISA) (Livzon Group Reagent Factory, Zhuhai, China). Positive tests were confirmed by HIV-1/2 WB (MP Biomedicals Asia Pacific Pte. Ltd., Singapore). Syphilis serology was determined through RPR (rapid plasma regain, RPR) (Livzon Group Reagent Factory, Zhuhai, China). Serum specimens which were positive by means of RPR (rapid plasma reagin assay, RPR) were retested by TPPA (treponema pallidum particle assay, TPPA) (Serodia, Japan). Subjects with serum positive for both TPPA and RPR were determined to be currently infected with syphilis. The presence of HCV antibody was tested by ELISA (enzyme-linked immunosorbent assay, ELISA) (Livzon Group Reagent Factory, Zhuhai, China).

### Statistical Analysis

Questionnaires were double-entered and then checked for accuracy using Epi Data software (Epi Data Association, Odense, Denmark, version 3.1). Descriptive analyses were conducted to describe the demographic characteristics, AIDS awareness, behavior characteristics, prevalence of HIV, syphilis and HCV. Categorical data were described and analyzed by frequency and Chisquare test. Odds ratios (OR) and their 95% confidence intervals (CI) were calculated in univariate analysis. Multivariate analysis was done using a forward multiple unconditional logistic regression model in order to determine adjusted odds ratios (aOR) for risk-factors related to HIV infection after adjusting effects of the some possible confounding or background variables. Variables in the multivariate analysis were selected based on variables which were marginally significant with P<0.20 in univariate analysis. Variables with P<0.05 were retained in the final multivariate logistic model in a forward stepwise manner. Probabilities for removal and entry of factors were set to 0.15 and 0.20. This statistical analysis was done by SPSS version 13.0 (Chicago, IL, USA).

## Results

### Demographic characteristics

A total of 463 eligible MSM was completed the survey. Demographic characteristics of participants are shown in Table 1. The majority of participants were Han ethnicity (97.2%). The ages of participants ranged from 16 to 78 years old, while most of the participants were 21 to 34 years old and accounted for 60.3%. The proportion of single in the marriage status was 73.7%. The percentage of participants who had education over 12 years was 41.0%. The proportion of homosexual orientation in sexual orientation was 54.2%.

**Table 1:** Demographic characteristics of MSM in Harbin, P. R. China.

Characteristics	Number (n = 463)	Proportion (%)
Nationality		
Han	450	97.2
Non-Han	13	2.8
Age (yrs.)		
≤20	45	9.7
21–34	279	60.3
≥35	139	30.0
Marriage status		
Single	341	73.7
Married	74	16.0
Cohabitation	15	3.2
Divorced/widowed	33	7.1
Education		
1–9 years	126	27.3
10–12 years	147	31.7
>12 years	190	41.0
Sexual orientation		
Homosexual orientation	251	54.2
Bisexual and heterosexual orientation	212	45.8

A total of eligible MSM was 463 in this survey.

### HIV-related knowledge and characteristics of sexual behavior

Table 2 illustrated the statistical results of HIV-related knowledge, characteristics of sexual behavior and the status of intervention in this survey. The awareness rate of AIDS related knowledge was adopted the definition of National Evaluation Framework [25]. While six of eight questions which participant answered were right, it was called as the awareness. Relatively high AIDS awareness rate (86.8%) was exhibited in this survey. The age of first homosexual behavior was predominant from 18 to 24 years old groups (57.5%), and the average age was 24.2 years old. The years of homosexual behavior from 1 to 5 years group were predominant (35.6%) in this survey. In the past 6 months, the average of male sexual partners was 3 and 7.3% of participants had 2–5 male sexual partners. The highest one had 100 partners.

**Table 2:** HIV-related knowledge and characteristics of sexual behavior.

Variable	Number(n = 463)	Proportion (%)
AIDS awareness	402	86.8
Age of first homosexual behavior (year)		
<18	47	10.2
18–24	266	57.5
≥25	150	32.3
Duration of homosexual behavior (years)		
0–1	135	29.2
1–5	165	35.6
5–10	75	16.2
>10	88	19.0
Number of homosexual partners in the past 6 months		
0–1	184	39.7
2–5	219	47.3
6–10	40	8.6
>10	20	4.4
Unprotected sex in the past 6 months	231	57.6
Anal sex with male sexual partners in the past 6 months	401	86.6
Oral sex with male sexual partners in the past 6 months	343	74.1
Female sex partners in past 6 months	143	30.9
Commercial sexual behavior	27	5.8
Drug abuse in the past 6 months	7	1.5
STD symptoms in the past year	76	16.4
Accepted intervention in the past year	270	58.3
HIV testing in the past year	245	52.9

A total of eligible MSM was 463 in this survey. AIDS: Acquired Immune Deficiency Syndrome; STD: Sexually Transmitted Disease; HIV: Human Immunodeficiency Virus.

Slightly more than half (57.6%) of MSM never or seldom used condoms when they had the sex activity, 86.6% had anal sex, and 74.1% had oral sex with male sexual partners in the past 6 months. A small proportion of the study population (30.9%) had heterosexual behavior, in which 62% failed to use condoms when they had sex with female sexual partners (not showed in Table 2). In the past 6 months, 5.8% purchased sex from male partners or sold sex to male partners. Percentage of 1.5 used drugs, only 2 persons had intravenous drug, who had shared needles with others in a drug use. Seventy six of 463 (16.4%) participants had sexually transmitted disease (STD) symptoms in the past years. Thirty-four of 463 (58.3%) participants accepted intervention services which contained one of them including free condoms, publicity material and company education.

### Infection status of HIV, syphilis and HCV

As shown in Table 3, the prevalence of HIV and syphilis was 9.5% (44/463) and 14.3% (66/463), respectively. The prevalence of HCV was 0.6% (3/463). The prevalence of co-infection of HIV and syphilis was 1.9% (9/463). In order to acquire risk factors which associated with HIV infection, univariate and multivariate analysis were done in this survey.

**Table 3:** The infections of HIV, Syphilis and HCV.

	Screening number	Positive number	Infection rate (%)
HIV	463	44	9.5
Syphilis	463	66	14.3
HCV	463	3	0.6
co-infevtion of HIV and syphilis	463	9	1.9

HIV: Human Immunodeficiency Virus; HCV: Hepatitis C Virus.

### HIV infection and risk factors by univariate analysis

The results of univariate and trend test analysis are displayed in Table 4. The risk of HIV infection was significant high in the ≥35 years old group (OR 4.7, 95% CI 1.1 to 20.8, P = 0.026) when compared to the ≤20 years old group. Different age groups had different risks of HIV infection. The odds ratios (HIV prevalence) were 1.0 (4.4%), 1.4(6.1%) and 4.7(18.0%), respectively, in the ≤20 years old group, the 21–35 years old group and the ≥35 years old group. The risk of HIV infection presented an increased trend accompanying with the increasing age (trend test, χ^2^ = 13.922, P<0.01). The participants whose marital status was cohabitation had a higher risk of HIV infection than those married (OR 6.9, 95% CI: 1.7 to 28.1, P = 0.011). Compared with the 1 year or less group in duration of homosexual behavior, the above 10 years group was 5.4 times high infection with HIV (OR 5.4, 95% CI 1.9 to 13.6, P = 0.001). The odds ratios (HIV prevalence) were 1.0 (4.4%), 1.8 (7.9%), 2.6 (10.7%) and 5.4 (19.3%), respectively, in the 0 to1 years group, the 1 to 5 years group, the 5 to 10 years group and the ≥10 years group. With the increasing duration of homosexual behavior, the risk of HIV infection significantly increased (trend test, χ^2^ = 13.581, P = 0.001). Compared with 0 to 1 homosexual partners in the past 6 months, the participants with 5 to 10 homosexual partners had a higher risk of HIV infection (OR 3.3, 95% CI 1.3 to 8.6, P = 0.025). However, the participants with over 10 homosexual partners were 5.6 times more likely to be infected with HIV (OR 5.6, 95% CI 1.9 to 17.1, P = 0.003). With the increasing number of sexual partners, the risk of HIV infection also showed an increasing trend (χ^2^ = 11.785, P = 0.001). Participants who had protected sex in the past 6 months were lower the risk of HIV infection than those who had unprotected sex (OR 0.4, 95%CI 0.2 to 0.8, P = 0.01). This result revealed that protected sex in the past 6 months was a protective factor of HIV infection.

**Table 4:** Factors associated with HIV infection among MSM by a univariate analysis.

Factors	HIV infection (%)	OR	95%CI	P-value	Trend Test	
					χ2	p-value
**Age (years)**						
≤20	4.4	1.0			13.922	0.000**
21–35	6.1	1.4	0.311–6.254	0.662		
≥35	18.0	4.7	1.07–20.762	0.026*		
**Marital status**						
Married	6.8	1.0			-	-
Single	9.1	1.4	0.518–3.677	0.418		
Cohabitation	33.3	6.9	1.692–28.145	0.011*		
Divorced/widowed	9.1	1.4	0.310–6.149	0.700		
**Education**						
>12 years	10	1.0			-	-
10–12 years	8.8	0.9	0.416–1.832	0.719		
1–9 years	9.5	0.9	0.443–2.027	0.889		
**Sexual orientation**						
Bisexual and heterosexual orientation	11.4	1.0			-	-
Homosexual orientation	8.8	1.3	0.699–2.554	0.379		
**Place source**						
Fixed friends or friend introduction	16.7	1.0			-	-
Bar and bath	10.5	0.6	0.156–2.196	0.683		
Park	11.5	0.7	0.143–3.190	0.934		
Comrade website or QQ group	7.5	0.4	0.107–1.559	0.366		
**Age of first homosexual behavior (years old)**						
≥25	8.0	1.0			-	-
18–24	10.2	1.3	0.638–2.647	0.470		
<18	10.6	1.4	0.456–4.109	0.560		
**Duration of homosexual behavior (years)**						
0–1	4.4	1.0			13.581	0.000**
1–5	7.9	1.8	0.680–4.975	0.224		
5–10	10.7	2.6	0.885–7.704	0.083		
>10	19.3	5.4	1.942–13.644	0.001**		
**Homosexual partners in past 6 months**						
0–1	7.1	1.0			11.785	0.001**
1–5	7.8	1.1	0.523–2.344	0.779		
5–10	20	3.3	1.261–8.573	0.025*		
≥10	30	5.6	1.857–17.110	0.003**		
**Commercial sexual behavior**						
No	8.9	1.0			-	-
Yes	18.5	2.3	0.830–6.449	0.163		
**Sexual history**						
No	9.9	1.0			-	-
Yes	3.7	0.4	0.047–2.655	0.498		
**STD symptoms in the past year**						
No	10.9	1.0			-	-
Yes	7.9	0.7	0.285–1.724	0.438		
**Syphilis**						
Negative	8.8	1.0			-	-
Positive	13.6	1.6	0.746–3.577	0.216		
**AIDS Awareness**						
No	9.8	1.0				
Yes	9.5	1.0	0.381–2.369	0.924		
**Accepted intervention in the past year**						
No	10.4	1.0			-	-
Yes	8.9	0.8	0.452–1.576	0.594		
**Protected sex in the past 6 months**						
No	13.0	1.0			-	-
Yes	5.3	0.4	0.173–0.812	0.01*		
**HIV testing in the past year**						
No	11.0	1.0			-	-
Yes	8.2	0.7	0.385–1.341	0.297		

* P<0.05; **P<0.01, HIV: Human Immunodeficiency Virus; STD: Sexually Transmitted Disease; QQ group: a chat tools.

There were no differences in education, sexual orientation, place source, age of first homosexual behavior, commercial sexual behavior, sexual history and STD symptoms in the past year in the different groups. In addition, syphilis, AIDS awareness, accepted intervention and HIV testing also showed no differences in the past year in the different groups.

### HIV infection and risk factors by multivariate analysis

Stepwise logistic regression model was used to perform multivariate analysis which explains relationships between some factors and HIV infection after adjusting effects of the some possible confounding or background variables. Ages, marital status, duration of homosexual behavior, number of homosexual partners in the past 6 months and commercial sexual behavior were retained in the final multivariate logistic model in a forward stepwise manner (P<0.20). The analyzed results of stepwise logistic regression model are summarized in Table 5. The results revealed that duration of homosexual behavior (aOR 1.1, 95% CI 1.04 to 1.13, P = 0.0002) was the independent risk factors for HIV infection. Commercial sexual behavior (aOR 4.0, 95% CI 1.2 to 13.7, P = 0.025) may be an independent risk factor for HIV infection. Protected sex (aOR 0.4, 95% CI 0.17 to 0.99, P = 0.04) was an independent protective factor for HIV infection.

**Table 5:** The results of HIV infection by logistic regression analysis.

	β	Wald χ^2^	P-value	OR	95% CI
Duration of homosexual behavior	0.767	13.24	0.0002	1.08	1.036–1.125
Commercial sexual behavior	1.398	5.052	0.025	4.047	1.196–13.691
Protected sex	0.885	4.042	0.044	0.413	0.174–0.987

Variables with P<0.2 in the univariate analysis, such as ages, marital status, duration of homosexual behavior, number of male sex partners in past 6 months and commercial sexual behavior, were retained in the final multivariate logistic model in a forward stepwise manner. Finally, three Variables such as protected sex, commercial sexual behavior and duration of homosexual behavior entered in the multivariate logistic model.

## Discussion

The prevalence of HIV among MSM, China had been increasing rapidly in the past few years in Harbin, P. R. China. The surveillance data among MSM indicated that the prevalence of HIV had increased dramatically from 1% in 2006 to 4.4% in 2008 in Harbin [23]–[24]. The prevalence of HIV was 9.5% in this survey, suggesting a marked increase in the HIV prevalence among MSM in Harbin in recent years.

In our survey, 86.8% of participants from MSM gave the 6 or over correct answers of AIDS knowledge and 58.3% of participants received intervention services. There was 57.6% of participants who never used or seldom used condoms with male sexual partners in the past 6 months, which was much higher than those in previous studies in Qingdao and Beijing [26]. The high AIDS knowledge awareness, high intervention coverage and the low proportion of protective sexual behavior happened simultaneously and presented the separation of knowledge and behavior [27]. It may be why a significant increase of HIV prevalence happened among MSM in recent years in Harbin. In addition, more sexual partners were common in this population, the average of male sexual partners was 3, the highest was 100 in this survey. More sexual partners would significantly increase the risk for HIV infection and accelerate the spread of HIV in MSM. Participants with 5 to 10 or ≥10 male sex partners in the past 6 months had higher risk for HIV infection than those with 0 to 1 male sex partners (OR 3.3, 95% CI 1.3 to 8.6, P = 0.025; OR 5.6, 95%CI 1.9 to 17.1, P = 0.003).

In our survey, with the growth of age, the risk of HIV infection increased gradually, the participants aged ≥35 had significantly increased the risk of HIV infection than those in aged ≤20 (OR 4.7, 95%CI 1.1 to 20.8, P = 0.026). At the same time, compared to the participants who had one year or less in duration of homosexual behavior, the MSM with over 10 years had a high risk for HIV infection (OR 5.4, 95% CI 1.9 to 13.6, P = 0.001) (aOR 1.1, 95%CI 1.04 to 1.13, P = 0.0002). Thus, our results indicated that along with the increase in duration of homosexual behavior, the risk of HIV infection also correspondingly increased in MSM.

Syphilis infection was also very popular in MSM population. According to our results of testing for syphilis, 14.3% of participants were infected with syphilis and 1.9% for co-infected with HIV and Syphilis in this survey. Syphilis infections significantly increased the risk of HIV infection. Some studies found that, after controlling for other risk factors, participants who had been infected with syphilis were more likely to be infected with HIV [28]. Thus, the strength of syphilis diagnosis and treatment should be effectively increased in MSM population.

Participants whose marital status was cohabitation were more likely to be infected with HIV, the risk of HIV infection was 6.9 times higher than that married (OR = 6.9, 95%CI 1.7 to 28.1, P = 0.011). We could speculate that participants whose marital status was cohabitation had not received the bondage of marriage and they were not very single-minded to his partner, and therefore they maybe had more risk behavior and more sexual partners than those married.

Commercial sexual behavior may be also an important risk factor. 5.8% of participants had commercial sexual behavior in this survey. The result of multivariate analysis showed that participants were more likely to be infected with HIV than those without commercial sexual behavior (aOR 4.0, 95%CI 1.2–13.7, P = 0.025). Because of making money in the nature of commercial sexual behavior, participants would provide sexual services to more commercial partners, and provid sexual service to not only man customers but also man. Thus the MSW (male sex workers, MSW) played a ”bridge„ and ”amplifier„ role in HIV/AIDS transmission between MSM and the general population [29]–[30]. Due to this kind of crowd were hidden and floated, it was hard to find them. Implementation of intervention in this population was more difficult than those in without commercial sexual behavior. Therefore, commercial sexual behavior may be also an important risk factor.

A small proportion (30.9%) of this survey population had been engaged in heterosexual sex, which was less than other research results. A previous survey from Shenzhen (China) showed that 68.5% of MSM had sex with the female sex partners in 2004 [31]. Another survey from Petersburg (Russia) showed that 79% of MSM had sex with women [32]. In this survey, 16% of MSM was married, these MSM had sex with not only men but also their spouses. Due to both social and secular pressure, marriage made it possible to maintain the relationships with men. The high risk of behaviors among this population imply that MSM might be bridge population for HIV transmission [33]. In the AIDS/HIV intervention, it was a noticeable problem how intervention was done in bisexual MSM.

It was worth being advertent that safety of sex activities in the past 6 months was a protective factor for HIV infection in this survey. Our results revealed that participants who had a protected sex in the past 6 months was lower at a risk of HIV infection than those in an unprotected sex (OR 0.4, 95%CI 0.2 to 0.8, P = 0.01) (aOR 0.4, 95%CI 0.17 to 0.99, P = 0.044). By now, it is widely believed that using condoms in a correct way and the most effective way of preventing AIDS in the world, and it was also proved in this survey. In our study, 57.6% of MSM population never used or seldom used condoms, the 30.9% of MSM having sex with females and 62.0% of those having unprotected sex in the past 6 months. It accelerated HIV transmitting from MSM to general population in Harbin, P. R. China. Thus, the propaganda about correct condom-using in MSM should be strengthened and increased in order to prevent HIV infection themselves in the future. Considering the risk factors of HIV infection among MSM population, comprehensive intervention are needed urgently, including not only education and promotion of condom-use, but also reducing the number of sexual partners and creating a single, committed relationships.

### Limitations

This survey is subject to several limitations. The data was examined retrospectively from a cross-sectional study and cannot prove exact causality. The snowball sampling was used in this survey, some selected biases may have influenced the results of this study. Owing to the subjects of survey came from a convenient sample of MSM who live in Harbin, P. R. China, the results may not be generalized to the greater population of MSM. The participates were approached and invited to in this study by outreach staff from non-governmental organizations which specifically serve the MSM community. Some MSM were unwilling to expose their identities to the publicity, which was called recessive crowd. Thus, they did not participate in our survey. As it involves personal privacy in the questionnaire, the participants may not have answered accurately. There would be some recall bias on some problems related to behaviors in the last 6 months or in the past year.

### Conclusion

The prevalence of HIV among MSM was rapidly increased in the past few years in Harbin, P. R. China. Our findings showed the high AIDS knowledge awareness, high intervention coverage and the low proportion of protective sexual behavior happened simultaneously in MSM community of Harbin. They presented the separation of knowledge and behavior. Considering the ”separation of knowledge and behavior„ in MSM in Harbin, we might further study to understand the reasons of such a separation, and take corresponding intervention measures. Cohabitation, duration of homosexual behavior and homosexual partners in past 6 months were important risk factors which associated with the HIV infection and commercial sexual behavior may be also an important risk factor. Participants who had protected sex in the past 6 months were at a lower risk of the HIV infection. Because it is widely believed that using condoms in a correct way is the most effective way of preventing AIDS in the world, we should strengthen propaganda and expand the usage of the condom by multi-sectoral collaboration in MSM in Harbin. Syphilis infection was also very popular in this population, and significantly increased the risk for being infected with HIV. Targeted, tailored, and comprehensive interventions are needed urgently to prevent HIV infection amongst MSM.
